# Novel drug resistance mechanisms and drug targets in *BRAF*-mutated peritoneal metastasis from colorectal cancer

**DOI:** 10.1186/s12967-024-05467-2

**Published:** 2024-07-09

**Authors:** Christin Lund-Andersen, Annette Torgunrud, Chakravarthi Kanduri, Vegar J. Dagenborg, Ida S. Frøysnes, Mette M. Larsen, Ben Davidson, Stein G. Larsen, Kjersti Flatmark

**Affiliations:** 1 Departments of Tumor Biology, Norwegian Radium Hospital, Oslo University Hospital, Montebello, 0310 Oslo, Norway; 2 Departments of Gastroenterological Surgery, Norwegian Radium Hospital, Oslo University Hospital, Oslo, Norway; 3 Departments of Pathology, Norwegian Radium Hospital, Oslo University Hospital, Oslo, Norway; 4 Institute of Clinical Medicine, University of Oslo, Oslo, Norway; 5 Department of Informatics, University of Oslo, Oslo, Norway

**Keywords:** Peritoneal metastasis, Colorectal cancer, Drug resistance, Therapeutic targets

## Abstract

**Background:**

Patients with peritoneal metastasis from colorectal cancer (PM-CRC) have inferior prognosis and respond particularly poorly to chemotherapy. This study aims to identify the molecular explanation for the observed clinical behavior and suggest novel treatment strategies in PM-CRC.

**Methods:**

Tumor samples (230) from a Norwegian national cohort undergoing surgery and hyperthermic intraperitoneal chemotherapy (HIPEC) with mitomycin C (MMC) for PM-CRC were subjected to targeted DNA sequencing, and associations with clinical data were analyzed. mRNA sequencing was conducted on a subset of 30 samples to compare gene expression in tumors harboring *BRAF* or *KRAS* mutations and wild-type tumors.

**Results:**

*BRAF* mutations were detected in 27% of the patients, and the *BRAF*-mutated subgroup had inferior overall survival compared to wild-type cases (median 16 vs 36 months, respectively, p < 0.001). *BRAF* mutations were associated with *RNF43*/RSPO aberrations and low expression of negative Wnt regulators (ligand-dependent Wnt activation). Furthermore, *BRAF* mutations were associated with gene expression changes in transport solute carrier proteins (specifically *SLC7A6*) and drug metabolism enzymes (*CES1* and *CYP3A4*) that could influence the efficacy of MMC and irinotecan, respectively. *BRAF*-mutated tumors additionally exhibited increased expression of members of the novel butyrophilin subfamily of immune checkpoint molecules (*BTN1A1* and *BTNL9*).

**Conclusions:**

*BRAF* mutations were frequently detected and were associated with particularly poor survival in this cohort, possibly related to ligand-dependent Wnt activation and altered drug transport and metabolism that could confer resistance to MMC and irinotecan. Drugs that target ligand-dependent Wnt activation or the BTN immune checkpoints could represent two novel therapy approaches.

**Supplementary Information:**

The online version contains supplementary material available at 10.1186/s12967-024-05467-2.

## Introduction

Colorectal cancer (CRC) is the second leading cause of cancer-related deaths worldwide, with metastatic progression being the main cause of CRC mortality. The peritoneum is the third most common site of metastatic colorectal cancer (mCRC) after the liver and lungs, and patients with peritoneal metastases have inferior prognosis and response to chemotherapy compared to other metastatic sites [1, 2]. In patients with limited peritoneal disease, cytoreductive surgery (CRS) followed by hyperthermic intraperitoneal chemotherapy (HIPEC) may be offered as a potentially curative treatment, but this treatment is associated with risk of complications, and the long-term outcome is variable. In this context, molecular analyses could provide information to help understand the biology behind disease aggressiveness and drug resistance, as well as to identify biomarkers and new therapeutic targets needed to improve treatment selection and develop new treatment options for patients with PM-CRC.

Broad explorative molecular studies in this field are scarce and often reported as part of larger studies of mCRC, with a low number of PM-CRC cases included. At the DNA level, mutations have typically been reported either as multi-gene profiling of small cohorts or as single-gene analysis of larger cohorts, generally, with limited statistical power and varying quality and detail regarding clinical information [3]. More recently, two comprehensive mutational studies of larger cohorts (250–350 cases) have been published [4, 5], but still, interpretation is hampered by lack of clinical data or limited gene analysis. In spite of shortcomings in existing studies, and in agreement with findings in mCRC in general, PM-CRC patients with tumors that have mutations in the *BRAF* oncogene have been identified as a subgroup with less favorable prognosis than *BRAF* wild-type cases. A few transcriptomic studies have been performed on a limited number of PM-CRC cases (n = 4–52 cases) [3, 6, 7], focusing on differences in gene expression between subgroups of CMS4 tumors [6], between PM-CRC and primary cancers, and between responders and non-responders to CRS-HIPEC [7]. However, analyses to unravel molecular consequences of mutational subgroups on transcriptional changes have not been performed in PM-CRC, specifically. In this work, we have performed a broad molecular analysis on the genomic and transcriptomic level on tumor samples from patients undergoing CRS-HIPEC for PM-CRC as part of a national Norwegian cohort, including clinical data, aiming to understand mechanisms of aggressive biology and therapy resistance, and to identify novel treatment strategies for patients with PM-CRC.

## Materials and methods

### Patients and treatment

Patients undergoing surgery for suspected or verified PM-CRC at the Norwegian Radium Hospital, Oslo University Hospital, where the National Treatment Unit for CRS-HIPEC in Norway is located, were eligible for inclusion. Between September 2001 and September 2020, informed consent was obtained from 313 of 607 eligible patients. Tumor tissue was not available in 65 patients (only blood samples were collected) and the collected specimen contained insufficient amount of tumor tissue in further 18 patients, resulting in a study population of 230 patients (Figure S1). Clinicopathological data was retrieved from the institutional peritoneal surface malignancy database. The study was approved by the Norwegian Ethics Committee (ID# s-07160b) and written informed consent was obtained from the patients. Peritoneal tumor distribution was classified according to the peritoneal cancer index (PCI), ranging from 0 to 39 [8]. Cytoreductive surgery (CRS) was performed with intension to remove all visible tumor lesions. Residual tumor after CRS was classified by the completeness of cytoreduction (CC) score (CC-0, no tumor; CC-1, tumor < 2.5 mm; CC-2, tumor 2.5-25 mm; CC-3, tumor > 25 mm [9]. HIPEC with mitomycin C (MMC; 35 mg/m^2^ in 0.9% saline) was administered in three fractions (50%–0 min, 25%–30 min, 25%–60 min) if CC-0 was obtained.

### Tumor sample processing

Fresh tissue samples were collected at the time of surgery, immediately snap-frozen and stored at − 80 °C until further processing. The tumor content was assessed in H&E-stained frozen sections (median 30%, 4–59%). The samples were homogenized and disrupted using TissueLyzer LT (Qiagen, Hilden, Germany). DNA and RNA was extracted using the AllPrep DNA/RNA/miRNA Universal Kit (Qiagen, Düsseldorf, Germany), automated with the use of QIAcube (Qiagen). DNA/RNA concentration and purity [DNA: median A_260/280_ = 1.8 (1.6–2.2), RNA: median A_260/280_ = 1.9 (1.0–3.9)] was measured using NanoDrop 2000 spectrophotometer (Thermo Fisher Scientific, Waltham, Massachusetts, USA) and Qubit fluorometer (Thermo Fisher Scientific).

### Targeted DNA sequencing

Targeted next-generation sequencing was performed using the PGM/Ion GeneStudio S5 system with either Ion AmpliSeq™ Cancer Hotspot panel v2 (HS, n = 94, 50 genes; single nucleotide variation (SNV)) or Oncomine Comprehensive panel v3 (Onc, n = 136, 161 genes; SNV, copy number variation (CNV), fusion genes) from Thermo Fisher Scientific. Variants, CNV and fusion genes were called and annotated using Torrent Suite Variant Caller/ANNOVAR based in-house pipeline [10] and Ion Reporter Software V.5.10 (Onc) (Thermo Fisher Scientific). The following filtering criteria were set to minimize inclusion of germline variants and false positives: synonymous, UCSC common SNPs, MAF > 0.002, ExAc > 0.002, likely benign/benign in ClinVar database, phred score < 20, p > 0.05 homopolymer regions ≥ 8. All reported variants were manually reassessed using Integrative Genomics Viewer (IGV). The median coverage of called variants was 2085, enabling detection of variants down to 1% allele frequency. Of the 137 cases subjected to fusion gene analysis, 9 cases were reported as “no call” as there was not enough evidence to determine if the fusion was present. Fusion genes were validated with “breakpoint” qPCR using PrimeTime Gene Expression Master Mix and primers (Supplementary file 1) from Integrated DNA Technologies, followed by sanger sequencing (Microsyth seqlab GmbH, Göttingen, Germany).

### mRNA sequencing

Tumor samples (n = 30) with mutations in *BRAF *(*n* = *10*), *KRAS *(*n* = *10*) or neither of these genes (WT, n = 10) were subjected to mRNA sequencing. Samples were selected upon the following criteria: sufficient tumor content [mean: 42% (25–50)], RNA purity (A_260/280_ and A_260/230_ > 1.8), RNA integrity numbers (RIN) > 7.5. RIN values were estimated with Bioanalyzer RNA 6000 Nano kit (Agilent Technologies, Santa Clara, California, USA). Total RNA was diluted to 100 ng/μL in 15 μL in sterile H_2_O, and mRNA sequencing libraries (paired end 2 × 75 bp) were prepared using the TruSeq Stranded mRNA protocol. The mRNA sequencing was performed on a NextSeq500 machine from Illumina (San Diego, California, USA), with a depth of 40–45 mill read pairs per sample.

### Transcript quantification and filtering

For transcript quantification, we used Salmon version 1.4.0 [11] in selective alignment mode with a decoy-aware transcriptome, which is known to mitigate potential spurious mappings arising because of sequence similarity of unannotated and annotated regions [12]. The default k-mer length of 31 was used for generating the transcriptome indices as the chosen k-mer size has been shown to work well with reads of 75 bp long [11]. The index was built on the transcriptome of genome reference consortium human build 38 patch release 13 (GRCh38p13) including alternative loci. Salmon's variational Bayesian EM algorithm was used for optimizing the abundance estimates. Salmon's built-in models to correct the sequence-specific biases, as well as fragment-level GC biases, were used. To increase the detection power [13] and confidence of the findings (particularly of lowly expressed genes), we filtered out genes with low/no expression before any subsequent analyses. For this, we followed a similar approach as described by Hebenstreit et al. [14]. Briefly, we performed a model-based clustering of the regularized log-transformed expression counts of all protein-coding genes in each sample into two classes using finite normal mixture models implemented in Mclust [15]. The two classes represent genes that are expressed and genes that have low/no expression. We imposed a further restriction that a gene has to be called as expressed in more than half of the samples within any of the included biological groups to be deemed expressed.

### Differential gene expression analysis, gene set enrichment analysis (GSEA) and consensus molecular subtype (CMS) classification

Differential expression analysis was performed to compare tumors with mutated *BRAF*, *KRAS* and WT using DESeq2 Bioconductor package version 1.36.0 [16]. Genes were deemed differentially expressed at a false discovery rate of 10% and absolute median log2 fold change > 1 (Figure S2). Validation of gene expression of selected genes was performed by qPCR using SsoAdvanced universal probes supermix and ready-made primePCR Probe Assay FAM 200R from Bio-Rad. GSEA was conducted using gProfiler (Elixir resources, [17]) where pathways from KEGG and Reactome databases were included. Consensus molecular subtype (CMS) classification was performed using single sample prediction in the CMSclassifier R package (Sage Bionetworks; 2022) provided by Guinney et al. [18] and the CMScaller [19]. Discrepancy between the two classifiers occurred in one case only, and the result from the CMSclassifier was reported in this case. For cases where one of the classifiers failed to call a subtype (NA, n = 7), the result from the other classifier was reported.

### Analysis of microsatellite instability (MSI)

Tumor MSI and MSS status was determined by analysis of tumor DNA using the Idylla™ MSI Assay (Biocartis, Mechelen, Belgium) according to the manufacturer’s instructions [20].

### Statistical analysis

To determine significant co-occurrence between *BRAF* or *KRAS* mutations and the other ten most frequently mutated genes (n > 13), a two-sided Fisher Exact test was performed (p < 0.05). For significant co-occurring mutations, the p-value was adjusted for multiple testing by using the Benjamini–Hochberg correction in R (p < 0.1). Two-sided Fisher Exact test was also used to determine co-occurrence between *BRAF*/*KRAS* mutations and fusion genes, in addition to associations between *BRAF* or *RNF43* mutations and right sided primary tumor. Power calculations for the RNA-sequencing experiment were performed using the “PROPER” method [21, 22]. Groups of 10 cases were found to be sufficient to detect differences in gene expression between the groups with 70% power.

The clinical data was analyzed with SPSS statistics (version 29.0.0.0 (241), IBM Corp, Armonk, NY). Variables are described with percentages or medians (min–max) unless stated otherwise. CC-scores were categorized into CC-0 or CC 1–3 (CC-1, CC-2, CC-3). Variables in these subgroups were compared using Chi-square and Pearson’s correlation for percentages and Mann–Whitney U for medians. Overall survival (OS) was defined as the time (in months) from the first procedure with the intention to perform CRS (index operation) to the date of death (from the Norwegian National Population Registry) or the censor date (June 1, 2022). Progression-free survival (PFS) was defined as the time (in months) from the index operation to the first recurrence of CRC, the last date of radiological imaging or death. The reverse Kaplan–Meier method was used to describe the follow-up time for OS and PFS. Univariable analyses were performed by the Kaplan–Meier method to estimate OS and PFS and compared by log-rank. A p-value of < 0.05 was considered statistically significant. Variables statistically significant in univariable analysis were included in multivariable analysis using Cox proportional hazards regression, in addition to gender, age and PCI (significant p < 0.1).

## Results

### Patients, surgical procedures, and long-term outcome

Of the 230 study patients, 158 were female (69%) and the median age was 61 years (range 22–80 years). pCRCs were located in the right colon (n = 109; 47%), the left colon (n = 81; 35%), the appendix (n = 22; 10%) and the rectum (n = 8; 8%), and TNM classification was available for 215 cases (Table 1). The majority of the patients had complete cytoreduction (n = 165; 72%). Of these, five patients did not receive HIPEC because of patient-related (n = 3) or practical (n = 2) reasons. The CC 1–3 patient group had a higher median PCI score compared to the CC-0 group, (CC score 26 and 10, respectively), otherwise, there were no significant differences in the patient characteristics between the groups (Table S1). The median follow-up time was 69 months (95% CI 62–76) for OS and 57 months (95% CI 52–62) for PFS. One-hundred-and-sixty-four patients died during follow-up, 104 (63%) and 60 (92%) in the CC-0 and CC 1–3 groups, respectively, resulting in a median OS of 43 months (34–52 months) and 14 months (7–21 months), respectively. One of the 165 patients in the CC-0 group was lost to follow-up, leaving 164 patients for assessment of recurrent disease, which was detected in 130 patients (80%). The estimated median PFS in the CC-0 group was 9 months (95% CI 7–12, Table 1). The peritoneum was the most frequent site of recurrence (84/130; 65%), with the peritoneum as the only site in 47 cases, and together with other metastatic sites in 37 cases. The first site of recurrence in the remaining 46 cases were in the form of liver metastases only (n = 16), lung metastases only (n = 11), and other or multiple sites (n = 19).

**Table 1:** Clinical parameters of the study cohort

Variable	Total, n (%)	CC-0, n (%)	CC 1–3, n (%)
Patients	230 (100)	165 (72)	65 (28)
Median age, years (min–max)^ns^	61 (22–80)	62 (25–76)	60 (22–80)
Gender^ns^			
Female	158 (69)	116 (70)	42 (65)
Male	72 (31)	49 (30)	23 (35)
Primary locations^ns^			
Appendix	22 (10)	13 (8)	9 (14)
Right colon	109 (47)	78 (47)	31 (48)
Left colon	81 (35)	60 (36)	21 (32)
Rectum	18 (8)	14 (9)	4 (6)
T-stage^ns^			
pT1	1 (0)	1 (1)	–
pT2	1 (0)	1 (1)	–
pT3	83 (36)	62 (38)	21 (32)
pT4	130 (57)	92 (56)	38 (59)
ND	15 (7)	9 (5)	6 (9)
N-stage^ns^			
pN0	59 (26)	48 (29)	11 (17)
pN1	90 (39)	64 (39)	26 (40)
pN2	68 (30)	49 (30)	19 (29)
ND	13 (6)	4 (2)	9 (14)
PCI score			
Median (min–max)^***^	12 (0–39)	10 (0–28)	26 (6–39)
0–10	87 (38)	84 (51)	3 (5)
11–20	79 (34)	67 (41)	12 (20)
21–30	44 (19)	14 (9)	30 (49)
31 and higher	16 (7)	0	16 (26)
ND	4 (2)	0	4
Performance status^ns^			
ECOG 0	188 (82)	137 (83)	51 (78)
ECOG 1	21 (9)	17 (10)	4 (6)
ECOG 2–4	5 (2)	4 (2)	1 (2)
ND	16 (7)	7 (4)	9 (14)
Long-term outcome			
OS median, months (95% CI)^***^	32 (28–36)	43 (34–52)	14 (7–21)
OS 5-years (%)	30	39	9
PFS median, months (95% CI), n = 164	–	9 (7–12)	–

Medians were compared between CC-0 and CC 1-3 using Mann–Whitney U; Frequencies were compared by chi-square; OS was compared by log-rank test

PCI: peritoneal cancer index; OS: Overall survival; PFS: progression free survival; ND: not determined; ns: not significant

^*^P < 0.05

^**^P < 0.01

^***^P < 0.001

^****^P < 0.0001

### Analysis of DNA aberrations

**Fig. 1:**
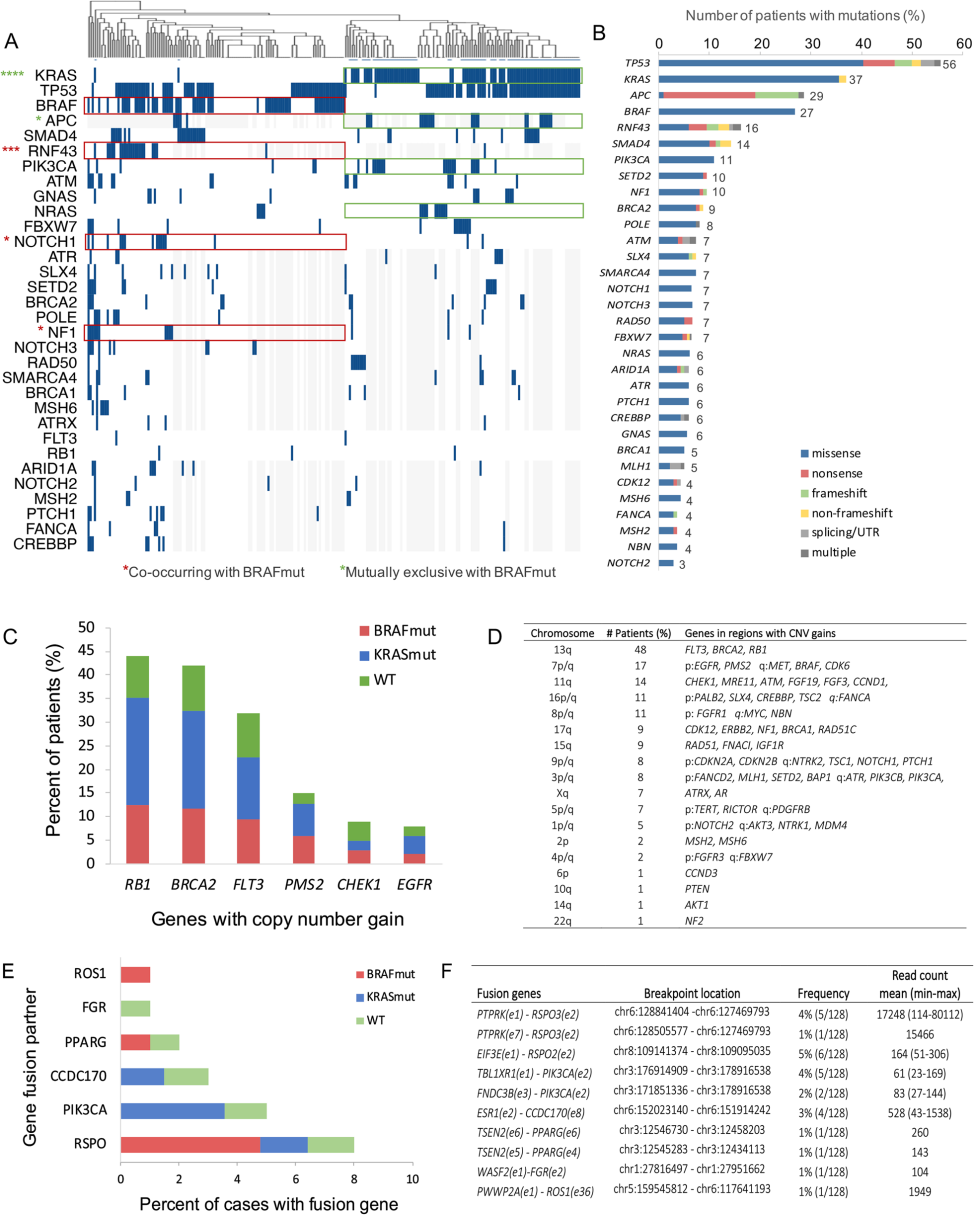
DNA aberrations in PM-CRC. **A** Genes mutated in PM-CRC patients (n = 230) detected by targeted DNA sequencing: unsupervised clustering of mutational profiles, blue marker: gene mutation, grey marker: data not available, red and green outline: co-occurring or mutually exclusive mutations before multiple testing, *p_adj_ < 0.1, **p_adj_ < 0.01, ***p_adj_ < 0.001, ****p_adj_ < 0.0001. *BRAF* mutations frequently co-occur with *RNF43*, *NOTCH1* and *NF1* mutations and are mutually exclusive with *KRAS* and *APC* mutations. **B** gene mutation frequency of genes mutated in more than five cases; colors indicate different mutation types. *BRAF* mutations are surprisingly frequent, present in 27% of the patients. **C** and **D** Copy number gains found in PM-CRC patients (n = 137). Colors indicate co-occurrence with mutations in *BRAF*, *KRAS* or wild-type (WT). **E** and** F** Fusion genes found in PM-CRC patients (n = 128) by targeted RNA sequencing, colors indicate co-occurrence with mutations in *BRAF*, *KRAS* or wild-type (WT). *BRAF* mutations frequently co-occur with R-Spondin (*RSPO*) fusions (p = 0.02), while *KRAS* mutations co-occur with *PIK3CA* fusions (p = 0.04)

Non-synonymous mutations were detected in 104 genes, and 32 genes (31%) were mutated in more than 4 patients (Fig. 1A and B, supplementary file 1/2). No mutations were detected in three cases (tumor content 30–50%), and in four cases only intronic mutations were found. The most frequently mutated genes were *TP53* (56%), *KRAS* (37%), *APC* (29%), *BRAF* (27%), *RNF43* (16%), *SMAD4* (14%) and *PIK3CA* (11%) (Fig. 1B), which all are commonly mutated in CRC [23]. The majority of the mutated genes had mainly missense mutations. The *BRAF* mutations were predominantly V600E, with two exceptions (K601E, I714V), altering the activation segment of the kinase domain and increasing kinase activity. *KRAS* was commonly mutated in codon 12 and 13 (79%: G12D (27%), G13D (19%), G12V (15%), G12S (8%), G12C (6%), G12A (2%), G12W (2%)), causing constitutive activation of RAS signaling. For *APC* and *RNF43,* frameshift and nonsense mutations were common, usually resulting in abnormal, non-functional proteins. The *RNF43* mutations were almost exclusively located in the extracellular (ECD) and RING finger domains (aa 1–317) of the protein, regions required for interaction and degradations of Frizzled (FZD), resulting in inhibition of WNT signaling [24]. The *APC* mutations were mainly located in the mutation cluster region (aa 1284–1580) of the β-catenin binding domain. By hierarchical clustering and statistical analysis, we found that *KRAS* mutations were mutually exclusive to *BRAF*, *RNF43* and *NRAS* mutations (p < 0.0001, p < 0.1, p < 0.1, Fig. 1A, Table S1). Furthermore, *RNF43*, *NOTCH1* and *NF1* mutations frequently co-occurred with *BRAF* mutations (39% vs 8%, p < 0.001; 15% vs 4%, p < 0.1; 19% vs 6%, p < 0.1 respectively, while *APC* mutations were mutually exclusive (35% vs 12%, p < 0.1)(asterisk in Fig. 1A, Table S2).

Copy number gains were detected in 59% (81/137) of the PM-CRC samples. Around half of these tumors had copy number gains (copy number > 4) in chromosome (Chr) 13q, in segments harboring the genes *FLT3* (32%), *BRCA2* (42%) and *RB1* (44%) (Fig. 1C and D, S3, supplementary file 3). Gains were also found in in Chr 7 (17%) of and Chr 11q (14%). Frequently occurring genes with copy number gains were equally distributed across PM-CRC mutational subgroups (*BRAF*mut, *KRAS*mut and WT, Fig. 1C).

Fusion transcripts were detected in 19% (24/128) of PM-CRC cases. The recurrent gene fusion partners included R-spondin (*RSPO2/3*, 8%), *PIK3CA* (5%), *CDC170* (3%) and *PPARG* (2%) genes that are located on Chr 3, 6, and 8 (Fig. 1E and F, S4, supplementary file 4). The *RSPO3*, *CCDC170*, *PPARG* and *ROS1* fusions were successfully validated with “breakpoint” qPCR and Sanger sequencing in some of the samples (Figure S5). The R-spondins are secreted proteins known to activate the canonical WNT signaling [25], and the *RSPO* fusions were found to be enriched in *BRAF* mutated tumors (p = 0.02, Fig. 1E). The *PIK3CA* fusions are known to result in overexpression of *PIK3CA,* driven by its fusion partners [26]. To our knowledge, *PIK3CA* fusions have not been detected in CRC previously, but have been described in breast cancer and two other cancer types. The *PIK3CA* fusions were found to be enriched in *KRAS* mutated tumors (p = 0.04, Fig. 1E).

Sixteen cases (7%) were microsatellite instable (MSI) (supplementary file 5), the majority co-occurring with *BRAF* mutations (9/16, p = 0.01). The remaining MSI cases (7/16) were almost equally distributed between *KRAS* mutated (n = 3) and WT cases (n = 4).

### *BRAF* mutations associated with poor long-term outcome

**Fig. 2:**
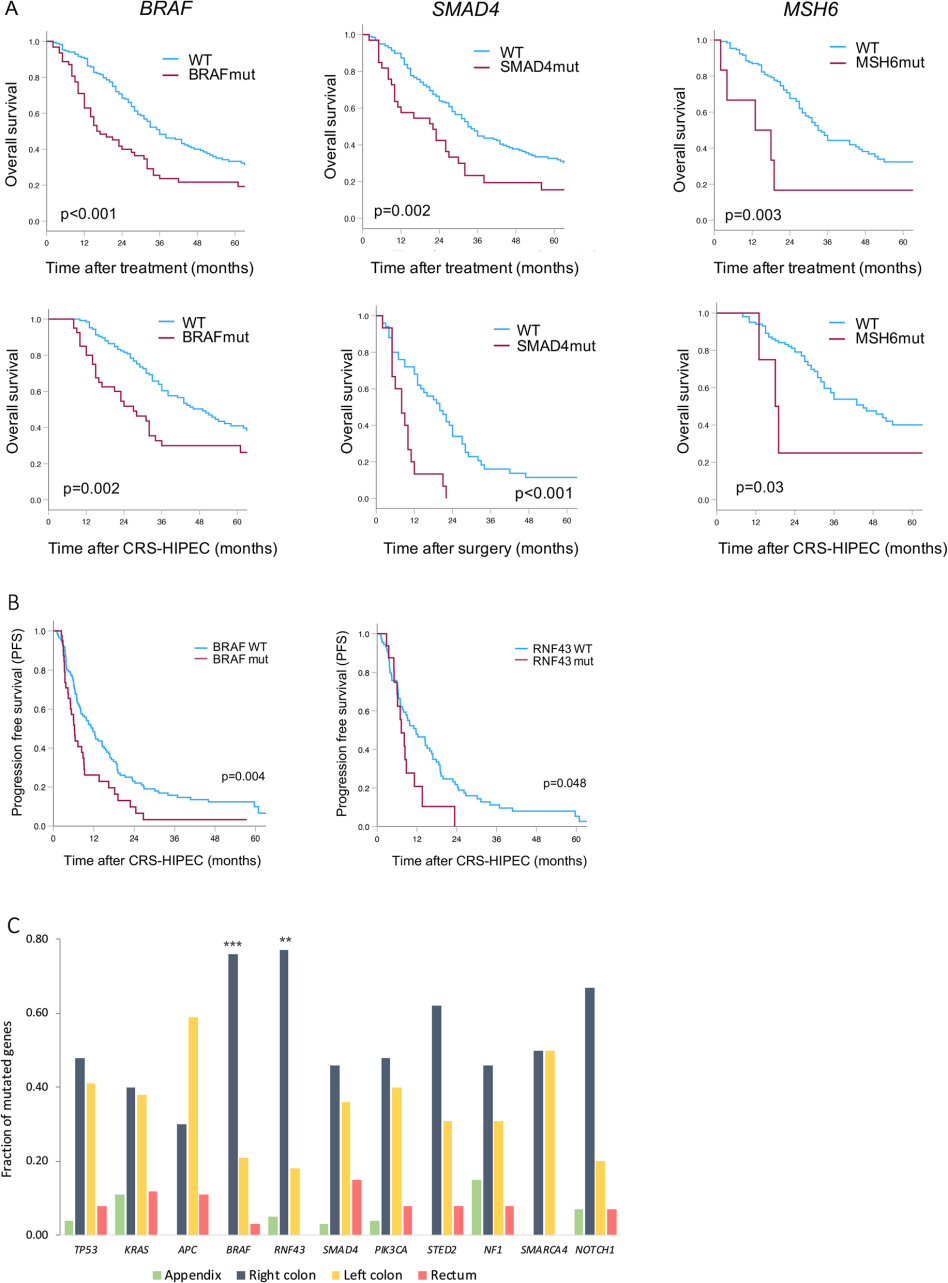
Associations between mutations and long-term outcome. **A** Significant findings from univariable analyses of overall survival (OS) for the total cohort (n = 230); mutated *BRAF*, *SMAD4* and *MSH6* compared to wild-type (WT) (upper panel) and for cohort subgroups; mutated *BRAF* (CC = 0), *SMAD4* (CC ≥ 1) and *MSH6* (CC = 0) compared to WT (lower panel). **B** Progression-free survival (PFS, n = 164). **C** Mutated genes associated with primary tumor location, *p < 0.05, **p < 0.01, ***p < 0.001, ****p < 0.0001. *BRAF* and *RNF43* mutations are associated with right sided primary CRC

In univariable analyses, *BRAF* mutation was strongly associated with inferior OS (median OS: 16 vs 36 mo, p < 0.001) and PFS (median PFS: 6 vs 12 mo, p = 0.004) compared to non-mutated cases. In addition, *SMAD4* and *MSH6* mutations were associated with inferior OS, and *RNF43* mutations were also associated with shorter PFS (Fig. 2A and B, Table S3/S4). In multivariable analysis *BRAF* (HR: 1.99) and *SMAD4* (HR: 1.57) mutations remained associated with inferior OS, in addition to PCI (HR: 1.09) and N2-stage (HR: 1.54) (Table 2). *MSH6* mutations were excluded due to small number of cases (n = 6). Factors associated with PFS in multivariable analysis were age (HR: 1.02), PCI (HR: 1.06) and *BRAF* mutations (HR: 1.51). *RNF43* mutations were excluded as they were only accounted for in a proportion of cases (106/164).

**Table 2:** Multivariable analysis of OS and PFS

Variable	OS (n = 230)		PFS (n = 164)	
	HR (95% CI)	p-value	HR (95% CI)	p-value
Age (increasing)	1.01 (0.99–1.03)	0.25	1.02 (1.01–1.04)	0.009
Gender (ref female)	1.29 (0.91–1.83)	0.15	1.29 (0.89–1.89)	0.184
PCI (increasing)	1.09 (1.07–1.12)	< 0.001	1.06 (1.03–1.09)	< 0.001
N2-stage	1.54 (1.06–2.23)	0.024	–	–
*BRAF* mutation	1.99 (1.39–2.83)	< 0.001	1.51 (1.01–2.26)	0.044
*SMAD4* mutation	1.57 (1.01–2.45)	0.045	–	–

### *BRAF* mutations associated with right-sided serrated primary CRC

**Fig. 3:**
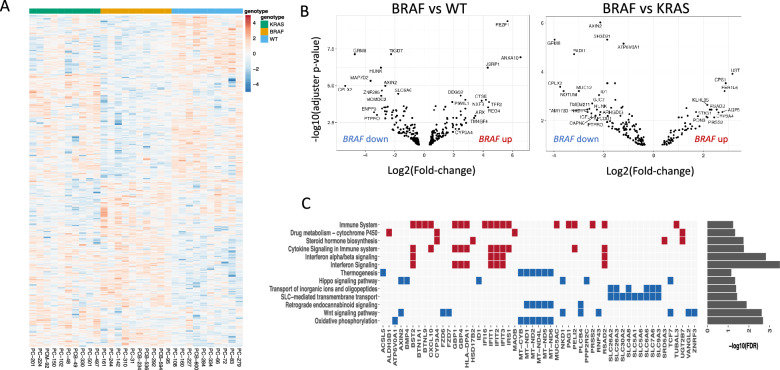
Differential gene expression and gene set enrichment analyses. **A** Heatmap of significant differential expressed genes (DEGs, p < 0.1) for individual patient samples comparing cases with mutated *BRAF *to mutated *KRAS* or WT, blue color: low expression, red color: high expression. Normalized counts from DESeq2 are used for visualization. Rows are centred and scaled. **B** Volcano plots showing DEGs significantly associated with *BRAF* mutation compared to WT (left panel) and *BRAF* mutation compared to *KRAS* mutation (right panel). **C** Gene set enrichment analysis showing affected signaling pathways and the DEGs involved, red markers: DEGs upregulated in *BRAF* mutated cases, blue markers: DEGs downregulated in *BRAF* mutated cases. Drug metabolism and immune signaling pathways are enriched in *BRAF* mutated cases, while SLC-mediated transport and Wnt signaling pathways are diminished

**Fig. 4:**
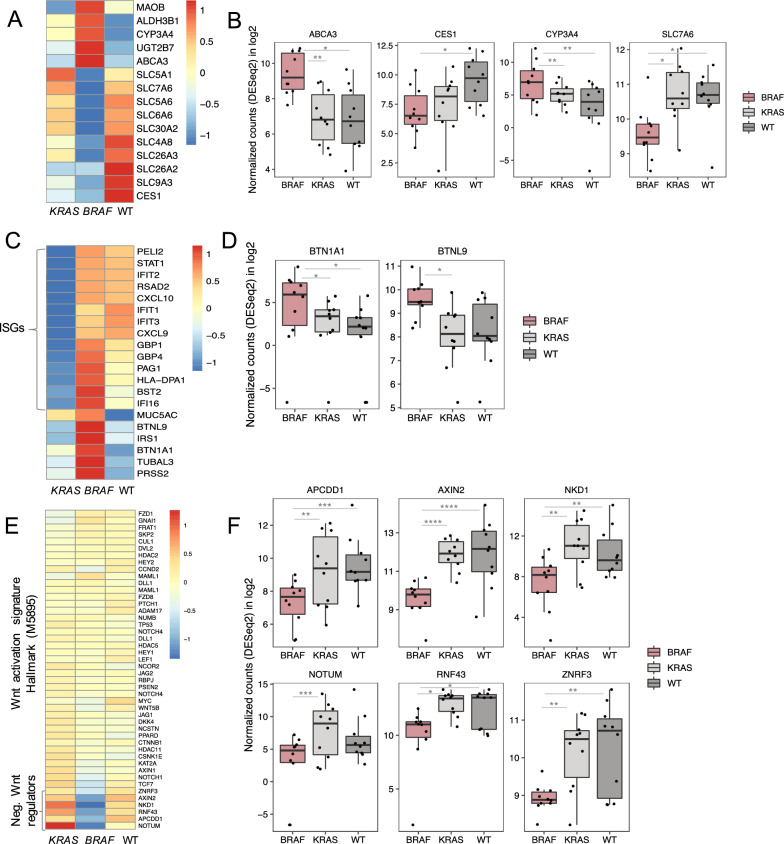
Differential expression of genes involved in drug metabolism, transport, immune signaling and Wnt signaling. Heatmaps with relative average gene expression levels within each group (*BRAF*, *KRAS*, WT) and boxplots of selected genes (indicating median, 25 and 75 percentile): **A** and **B** Altered expression of drug metabolism genes e.g. *CYP3A4* and *CES1* known to metabolize irinotecan, and reduced expression of *SLC* genes (e.g. *SLC7A6*) involved in transport of mitomycin C in *BRAF* mutated cases. **C** and **D** Increased expression of interferon stimulated genes (ISG, compared to *KRAS* mutated only) and BTN checkpoint molecules in *BRAF* mutated cases. **E** and **F** Similar expression of genes involved in the Wnt activation signature (Hallmark M5895) between the groups, however reduced expression of negative Wnt regulators in *BRAF* mutated cases, associated with ligand-dependent Wnt signaling. *p_adj_ < 0.1, **p_adj_ < 0.01, ***p_adj_ < 0.001, ****p_adj_ < 0.0001. Mean normalized read counts (DESeq2) in log2 scale were centred and scaled for visualization in heatmaps (**A**, **C**, **E**)

Differential gene expression analysis of *BRAF* mutated versus *KRAS* mutated and WT cases, revealed 179 and 303 differentially expressed genes (DEGs), respectively (p_adj_ < 0.1, up-regulated: 63 and 140, down-regulated: 116 and 163, Fig. 3A and B, supplementary file 6). The top ten up and down-regulated genes within each comparison are listed in Table S5 and S6. Among the top up-regulated genes in *BRAF* mutated compared to WT cases were *ANXA10* (Annexin A10), *TFF1* (Trefoil factor 1), *TFF2* (Trefoil factor 2) and *CTSE* (Cathepsin E), which are all markers associated with *BRAF* mutated right sided sessile serrated primary CRC [27–30]. *LY6G6D* (lymphocyte antigen-6 family member G6D) was found heavily down-regulated in *BRAF* mutated cases compared to WT**,** a feature that is also associated with promoter hypermethylation and sessile serrated polyps [31]. Together with our findings of down-regulated *RNF43* and *ZNRF3* in *BRAF*-mutated PM-CRC (Figs. 3C, 4E and F, [32]) and enrichment of *BRAF* and *RNF43* mutations in right sided primary CRC cases (Fig. 2C, supplementary file 7), previous findings that the *BRAF*-mutated PM-CRC are associated with right-sided serrated primary CRC are confirmed.

### GSEA—Altered drug metabolism and transport in *BRAF*-mutated PM-CRC

GSEA of DEGs identified down-regulation of a range of SLC (solute carrier) transmembrane transporters in *BRAF-*mutated cases (Fig. 3C, Table S7). Expression of these transporters, which are important for the uptake of key cytotoxic drugs, was reduced compared to *KRAS*-mutated and WT cases (*SLC5A1*, *SLC6A6*, and *SLC7A6*), and compared to WT only (although a trend was also seen compared to *KRAS*-mutated cases (*SLC30A2*, *SLC5A6*, *SLC26A3*, *SLC26A2*, *SLC9A3*, *SLC4A8*), Fig. 4A and B). Of particular interest in this cohort was down-regulation of *SLC7A6* (validated: Figure S6) which is associated with uptake of MMC, used in HIPEC treatment of the patients in this study [33]. In addition, another transporter that regulates drug efflux from the cells [34], the ATP-binding Cassette transporter *ABCA3,* was over-expressed in the *BRAF-*mutated cases compared to the other subgroups (Fig. 4B and Table S6). *ABCA3* expression is associated with poor survival and multidrug resistance in Leukemia cells [35] and may have similar functions in *BRAF-*mutated colorectal cancer.

GSEA also revealed up-regulation of genes involved in drug metabolism, including irinotecan metabolism, in *BRAF*-mutated cases versus WT (Fig. 3C, Table S7): *CYP3A4* (Cytochrome P450 enzymes 3A4, validated: Figure S6)*, UGT2B7* (UDP-Glucuronosyltransferase-2B7), *MAOB* (Monoamine Oxidase B) and *ALDH3B1* (Aldehyde Dehydrogenase 3 Family Member B1). *CYP3A4* was also found to be elevated in *BRAF*-mutated cases compared to *KRAS*-mutated (Fig. 4A and B). In addition, CES1 (Carboxylesterase 1) was down-regulated in the *BRAF*-mutated cases compared to WT.

### Immune signaling in *BRAF*-mutated PM-CRC

Genes that play a role in the immune system were up-regulated in *BRAF*-mutated cases (Fig. 3C). The interferon (IFN)-stimulated genes *RSAD2* (radical S-adenosyl methionine domain containing 2)*, GBP1* and *GBP4* (Guanylate Binding Protein 1 and 4)*, BST2* (Bone Marrow Stromal Cell Antigen 2)*, IFIT1, IFIT2 and IFIT3* (interferon-induced protein with tetratricopeptide repeats 1, 2 and 3)*, HLA-DPA1* (Major Histocompatibility Complex, Class II, DP Alpha 1), *CXCL10* (C-X-C motif chemokine ligand 10) and *IRS1* (Insulin receptor substrate-1) were enriched compared to the *KRAS-*mutated subgroup (Fig. 4C). Moreover, the immune checkpoint molecule *BTN1A1* (Butyrophilin subfamily 1 member, validated: Figure S6) was found up-regulated in *BRAF*-mutated cases compared to the other two subgroups (Fig. 4C and D). *BTNL9* (Butyrophilin-Like 9, validated: Figure S6) was also significantly higher expressed in *BRAF-*mutated compared to *KRAS*-mutated tumors, and a similar trend was seen for WT cases. The gel-forming mucin *MUC5AC* was also found to be highly expressed in *BRAF* mutated cases compared to the two other subgroups, although only significant towards *KRAS* mutated cases.

**Fig. 5:**
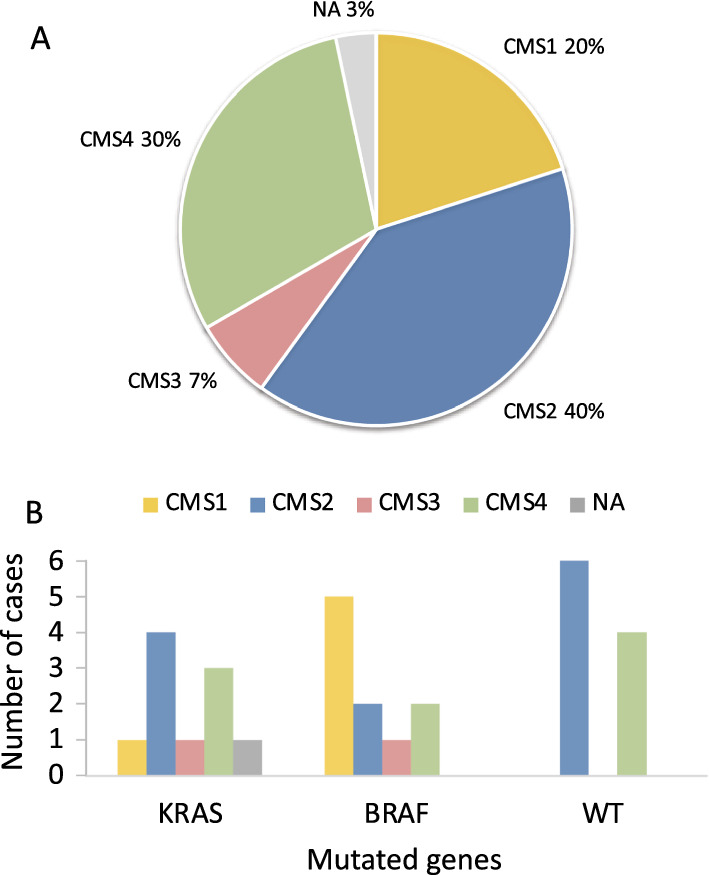
Consensus molecular subtype (CMS) classification. **A** Distribution of CMS subtypes in PM-CRC (n = 30). CMS1 and CMS4 are enriched compared to pCRC. **B** Distribution of CMS subtypes in PM-CRC mutational subgroups (*KRAS*, *BRAF*, WT). *BRAF* mutated cases are enriched with CMS1

The PM-CRC gene expression data was classified according to the colorectal consensus molecular subtypes, resulting in 40% CMS2 (canonical), 30% CMS4 (mesenchymal), 20% CMS1 (immune), 7% CMS3 (metabolic) and 3% unclassified (NA) (Fig. 5A, supplementary file 8). The *BRAF*-mutated cases were enriched with the immune subtype, CMS1, while *KRAS*-mutated and WT cases contained a mixture of CMS2 and CMS4 (Fig. 5B).

### GSEA—ligand-dependent WNT activation in *BRAF*-mutated PM-CRC

GSEA revealed reduced expression of genes involved in Wnt signaling in *BRAF*-mutated cases compared to *KRAS-*mutated and WT (Fig. 3C, Table S7). To investigate whether the Wnt pathway was less activated in *BRAF*-mutated cases, we applied the hallmark Wnt activation signature (MSigDB M5895) on our data, and found broadly similar gene expression levels across the subgroups, indicating equal Wnt activation (Fig. 4E). The discrepancies lay mainly within the negative Wnt regulators and down-regulation of *RNF43* and *ZNRF3,* located at the cell surface, and *AXIN2* (validated: Figure S6), *NKD1, APCDD1,* and *NOTUM,* involved in negative feedback regulation, in the *BRAF*-mutated cases compared to the other subgroups (Fig. 4E and F). The low expression of negative feedback regulators is previously associated with ligand-dependent Wnt signaling in *RNF43/RSPO* aberrated CRC [36], and consistent with our findings that *BRAF* and *RNF43* mutations/RSPO fusions often co-occur. Reduced expression of *RNF43* is also in line with the presence of nonsense and frameshift mutations (Fig. 1B).

## Discussion

In this cohort of PM-CRC cases, *BRAF* and *RNF43* were 3–8 times more frequently mutated (27% and 16%, respectively) compared to previous reports from analyses of liver, 9% and 3%, and lung metastases, 6% and 2%, respectively [23, 37]. In contrast, the *APC* mutation frequency (29%) was low compared to previous reports from primary CRC (75%), colorectal liver metastases (82%), and lung metastases (86%) [23]. *BRAF* and *RNF43* mutations frequently co-occurred and were associated with right-sided serrated primary tumors, in line with previous studies in CRC [32]. The differences observed between the metastatic locations suggest that the combination of *BRAF* and *RNF43* mutations are associated with metastasis to the peritoneal cavity. *BRAF* mutations in CRC are associated with more aggressive disease and poor outcome through associations with pathological features (poorly differentiated tumors, tumor budding), advanced disease stage at the time of diagnosis, and peritoneal metastasis [38]. In vitro*, BRAF* mutations have been connected to enhanced ability of migration and invasion of CRC cell lines [39]. *RNF43* mutations have also been associated with aggressive tumor biology, and in *BRAF* mutated patient derived organoids, *RNF43* mutations were recently suggested to have a key role in promoting metastasis in animal models [40, 41]. Taken together, the marked differences between the metastatic sites with high abundance of *BRAF* and *RNF43* mutations in PM may contribute to explain the inferior survival in PM-CRC.

In addition to the inherently aggressive biology of *BRAF*-mutated CRC, poor response to anti-cancer therapy could contribute to poor OS. MMC was the drug used for HIPEC in this study, and sensitivity to MMC would therefore be a key requirement for HIPEC efficacy. Our findings revealed down-regulation of several SLC transmembrane transporters in *BRAF-*mutated tumors. These molecules regulate uptake of cytotoxic drugs [33], and of particular interest, *SLC7A6*, which regulates uptake of MMC was strongly down-regulated in *BRAF*-mutated cases. To instigate cell killing, MMC must be taken up by the tumor cells, and reduced cellular uptake could therefore impair the efficacy of HIPEC. If validated on the protein level and through functional studies, this finding would suggest that other drugs should be considered for HIPEC in *BRAF*-mutated cases. The *BRAF*-mutated tumors also exhibited increased expression of several metabolic enzymes involved in the intracellular processing of anti-cancer drugs, which may lead to drug resistance and poor clinical efficacy. Another key drug in the management of mCRC is the topoisomerase1-inhibitor irinotecan. Irinotecan is converted to its active form (SN-38) by the intracellular enzyme CES1 [42], which was markedly down-regulated in the *BRAF*-mutated cases. In parallel, CYP3A4, another key enzyme which inactivates irinotecan, was up-regulated in the *BRAF*-mutated subgroup [42, 43], further potentially contributing to irinotecan resistance. Because follow-up after CRS-HIPEC is administered locally, details regarding irinotecan administration to patients in this study were not available. However, based on current oncological management of mCRC, it is reasonable to assume that a large proportion of patients were offered irinotecan-containing therapy as part of subsequent palliative systemic treatment for recurrent disease. Taken together, our findings reveal molecular changes pointing towards potential novel resistance mechanisms pertaining to two commonly administered drugs to patients with PM-CRC. These findings also provide a strong argument for determining the mutational status of *BRAF* early in the course of PM-CRC treatment, and ideally up-front of CRS-HIPEC.

Targeting *BRAF V600E-*mutated CRC using BRAF inhibitors alone has not been very effective, likely due to feedback activation of MAPK signaling through EGFR [44]. However, the BEACON clinical trial showed prolonged survival for mCRC patients when treated with BRAF inhibitors in combinations with EGFR and MEK inhibitors [45]. Although this treatment strategy could be an option for *BRAF*-mutated PM-CRC, caution should be taken as CYP3A4, discussed above, is also known to metabolize several BRAF and EGFR inhibitors, such as Vemurafenib [46], Encorafenib [47] and Erlotinib [48], and might reduce the efficacy of the drugs.

In contrast to pCRC, where ligand independent (Li) activation of the Wnt signaling pathway is dominant (in 85% of cases) mainly due to *APC* mutation [36], ligand-dependent (LD) Wnt activation was the principal mode of Wnt activation in the *BRAF*-mutated subgroup. At the genomic level, *BRAF* and *APC* mutations were almost mutually exclusive in our cohort, while *BRAF* mutations frequently co-occurred with *RNF43* mutations or RSPO fusions that are dependent on Wnt ligand for Wnt activation. In addition, low expression of negative Wnt regulators associated with LD activation [36], were found in all *BRAF*-mutated cases subjected to transcriptome analyses, including when *RNF43*/RSPO aberrations were not detected. These findings suggest that *RNF43*/RSPO aberrations are more commonly co-occurring with *BRAF* mutations than was documented in our study. Collectively, these results point to the possibility of targeting LD-Wnt signaling in *BRA*F-mutated PM-CRC. With the target present in the cell membrane, LD signaling is thought to be more easily “druggable” than Li-Wnt activation, where the target is located intracellularly [49]. A class of drugs that are being extensively explored in this context are the porcupine inhibitors (e.g. LGK974), which prevent secretion of the Wnt ligand from signaling cells. Such inhibitors have been effective in in vitro and in vivo models with *RNF43* and *RSPO* aberrations [49] and are currently being investigated in several clinical trials (NCT01351103, NCT03447470, NCT03507998). Interestingly, low or absent *AXIN2* expression, which we found to be reduced in *BRAF*-mutated cases, has been suggested as a biomarker for selecting patients for treatment with porcupine inhibitors [36]. Hence, these results suggest that targeting LD-Wnt signaling could be beneficial in *BRAF*-mutated PM-CRC, possibly using *AXIN2* expression as a biomarker for treatment selection which is more feasible than finding RNF43/RSPO aberrations through RNA-sequencing.

The majority of tumors in this cohort were microsatellite stable (MSS), with only 7% being MSI, which is in line with previous findings in mCRC (5–7% MSI cases) [50]. We and others have suggested that the negative prognostic impact of *BRAF* mutations could be counteracted by MSI status [51, 52], but although MSI cases were enriched within the *BRAF*-mutated subgroup, they still accounted for only 15% of the cases. Based on the frequency of MSI cases, immune checkpoint inhibitors currently in clinical use therefore do not seem to be an obvious treatment option in *BRAF*-mutated PM-CRC. In this context, the strong up-regulation of the newly discovered immune checkpoint molecules *BTN1A1* and *BTNL9* is very interesting. Although the T cell receptor for these molecules is still unknown, studies have shown that they inhibit CD4^+^ and CD8^+^ T cell proliferation and reduce production of IL-2 [53, 54]. An antibody against BTN1A1 (hSTC810) is already being studied in clinical trials (NCT05231746) [55] and if efficacious, BTN1A1 could represent a novel immunotherapy target in *BRAF-*mutated PM-CRC. The increased immune signaling in *BRAF-*mutated PM-CRC is also in line with enrichment of the immune subtype CMS1 in the *BRAF*-mutated cases. Collectively, these results indicate that *BRAF-*mutated PM-CRC have persistent immune signaling leading to increased levels of BTN immune checkpoint molecules that should be further explored as a possible novel therapeutic target for this particular patient subgroup.

A challenge, which is relevant in most studies when consecutive biobanking of surgical specimens is involved, was the inability to retrieve data from all eligible patients in this national cohort, as we ended up reporting data from 230 of 607 patients operated for PM-CRC. Prior to 2013, biobanking was anecdotal, while from 2013, a conclusion could be reached for more than half of eligible patients. In many cases, surgeons failed to collect tissue, the tissue tumor content was inadequate, or the sample failed subsequent quality control. There was no bias related to patient consent, but patients with low tumor burden and good response to neoadjuvant treatment may have been less likely to have their tumors sampled for research purposes (the surgeon prioritizing routine hisptopathology), and the samples would also be less likely to contain sufficient tumor tissue for subsequent analysis. Thereby, the mutational profiles could in principle be more representative of patients with high-volume disease and inferior chemotherapy response than of cases with very low tumor burden. Another limitation is related to the use of two targeted panels for mutation analysis, because a broader gene panel became available during the time period when the analyses were conducted, and the mutation status of some genes was therefore less extensively characterized (94/230 cases). The study cohort included patients with PM-CRC undergoing CRS and MMC-based HIPEC. While CRS is still standard of care for low-volume resectable PM, the use of HIPEC remains controversial after the failure of oxaliplatin-based HIPEC to improve outcomes in a randomized trial in oxaliplatin-pretreated patients [56]. MMC-based HIPEC has not been similarly studied in a randomized trial, and its value is thereby not fully clarified; however, our data would suggest that efficacy may be inferior in *BRAF*-mutated cases.

Overall, this study shows that *BRAF* mutations are frequent in PM-CRC, often co-occurring with *RNF43* or RSPO aberrations. This combination of abnormalities could lead to a more aggressive phenotype that partly may explain the particularly poor prognosis associated with *BRAF* mutations. Another contributor to poor prognosis may be altered drug metabolism and transport causing resistance to anti-cancer drugs MMC and irinotecan. Two potential novel therapeutic approaches were identified, suggesting the use of inhibitors to target LD-Wnt activation and specific targeting of the BTN immune checkpoints.

### Supplementary Information

Supplementary Material 1.

Supplementary Material 2.

Supplementary Material 3.

Supplementary Material 4.

Supplementary Material 5.

Supplementary Material 6.

Supplementary Material 7.

Supplementary Material 8.

Supplementary Material 9.

Supplementary Material 10.

## Data Availability

All data that supports the findings of this study and that do not compromise the privacy of research participants, are available as supplementary data. Sensitive data, such as raw sequencing and clinical data, are stored in the European Genome-Phenome Archive (EGA) data repository (accession number: EGAD50000000593).

## Abbreviations

PM-CRC: Peritoneal metastasis from colorectal cancer
HIPEC: Hyperthermic intraperitoneal chemotherapy
MMC: Mitomycin C
CRC: Colorectal cancer
mCRC: Metastatic colorectal cancer
CRS: Cytoreductive surgery
PCI: Peritoneal cancer index
SNV: Single nucleotide variation
CNV: Copy number variation
HS: Ion AmpliSeq™ Cancer Hotspot panel
Onc: Oncomine Comprehensive panel v3
IGV: Integrative Genomics Viewer
GSEA: Gene set enrichment analysis
CMS: Consensus molecular subtype
OS: Overall survival
PFS: Progression-free survival
MSI: Microsatellite instable
MSS: Microsatellite stable
HR: Hazard ratio
WT: Wild-type
DEGs: Differentially expressed genes
