# Supplementary material for: Novel drug resistance mechanisms and drug targets in BRAF-mutated peritoneal metastasis from colorectal cancer
Source: J Transl Med. 2024 Jul 9;22:646. doi: 10.1186/s12967-024-05467-2 (PMC11234641; doi:10.1186/s12967-024-05467-2)
Supplement: Supplementary file 10 — Supplementary Material 10. [file 12967_2024_5467_MOESM10_ESM.pdf]

Supplementary tables and figures

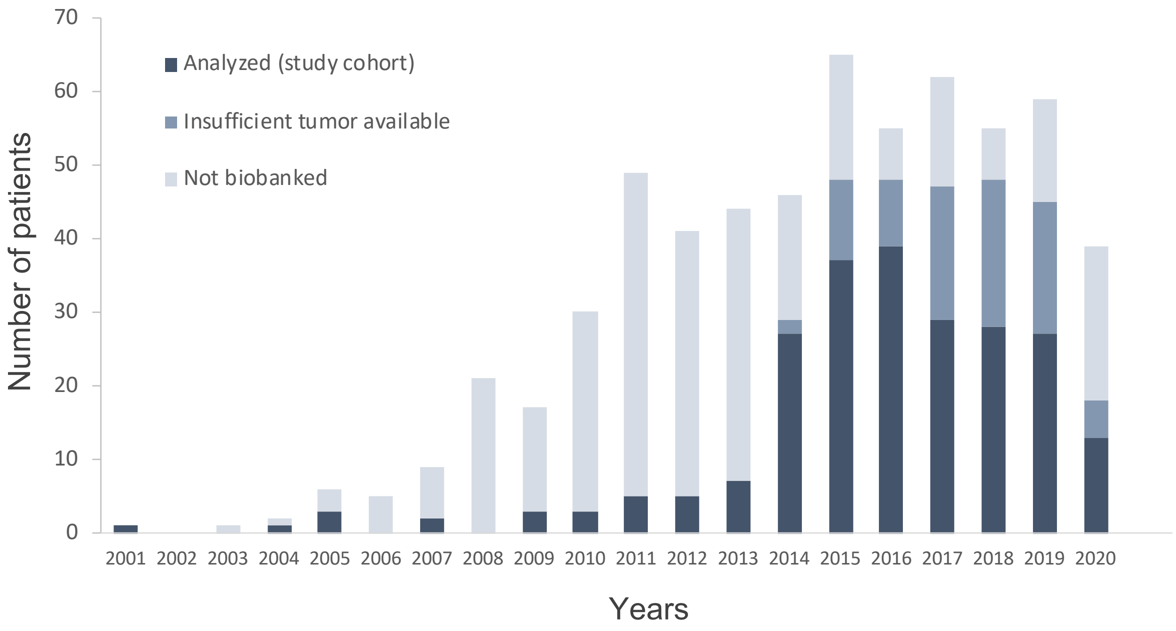

***Figure S1**: Number of patients undergoing surgery for PM-CRC at the Norwegian Radium Hospital, Oslo University Hospital per year between 2001 and 2020 (n=607). Biobanking was limited up until 2013, and hampered in 2020 due to the Coronavirus pandemic. Dark blue bars: cases analyzed and included in the study cohort (n=230), mid blue bars: cases biobanked (n=83) but not included as tumor tissue was unavailable (n=65) or present in insufficient amounts (n=18), light blue bars: eligible cases not biobanked (n=294)*

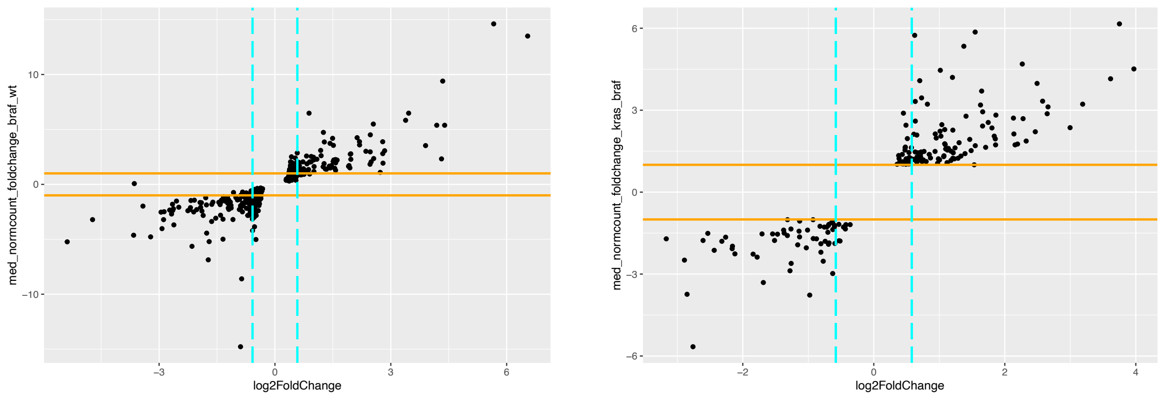

***Figure S2:** Genes were found differentially expressed at a false discovery rate of 10% and absolute median log2 fold change >1. The median log2 fold change (on y-axes) for both comparisons (left and right panels) was used for selecting genes instead of the standard log2 fold change (on x-axes) to minimize type II errors that may arise as a consequence of a few outlier values. The horizontal and vertical lines indicate the log2 fold change threshold of 1.*

Table S1: Genes co-occurring or mutually exclusive with *KRAS* mutations

Gene	mut/*KRAS* WT	mut/*KRAS*mut	p-value (FET)	p_{adj} (BH)
NOTCH1	0.09	0.02	0.056	1.07E-01
TP53	0.54	0.59	0.494	6.80E-01
RNF43	0.22	0.04	**0.012**	**4.00E-02**
PIK3CA	0.08	0.16	**0.048**	1.07E-01
BRAF	0.42	0.01	**6.23E-14**	**6.23E-13**
NF1	0.11	0.07	0.544	6.80E-01
APC	0.20	0.40	0.064	1.07E-01
NRAS	0.10	0.00	**0.003**	**1.50E-02**
SETD2	0.09	0.11	0.759	8.43E-01
SMAD4	0.14	0.15	0.846	8.46E-01

Table S2: Genes co-occurring or mutually exclusive with *BRAF* mutations

Gene	mut/*BRAF* WT	mut/*BRAF*mut	p-value (FET)	p_{adj} (BH)
NOTCH1	0.04	0.15	**0.006**	**2.00E-02**
TP53	0.52	0.65	0.134	1.68E-01
RNF43	0.08	0.39	**6.29E-05**	**3.15E-04**
PIK3CA	0.13	0.05	0.095	1.58E-01
KRAS	0.50	0.02	**6.23E-14**	**6.23E-13**
NF1	0.06	0.19	**0.041**	**8.20E-02**
APC	0.35	0.12	**0.024**	**6.00E-02**
NRAS	0.08	0.02	0.12	1.68E-01
SETD2	0.14	0.08	0.328	3.64E-01
SMAD4	0.13	0.18	0.399	3.99E-01

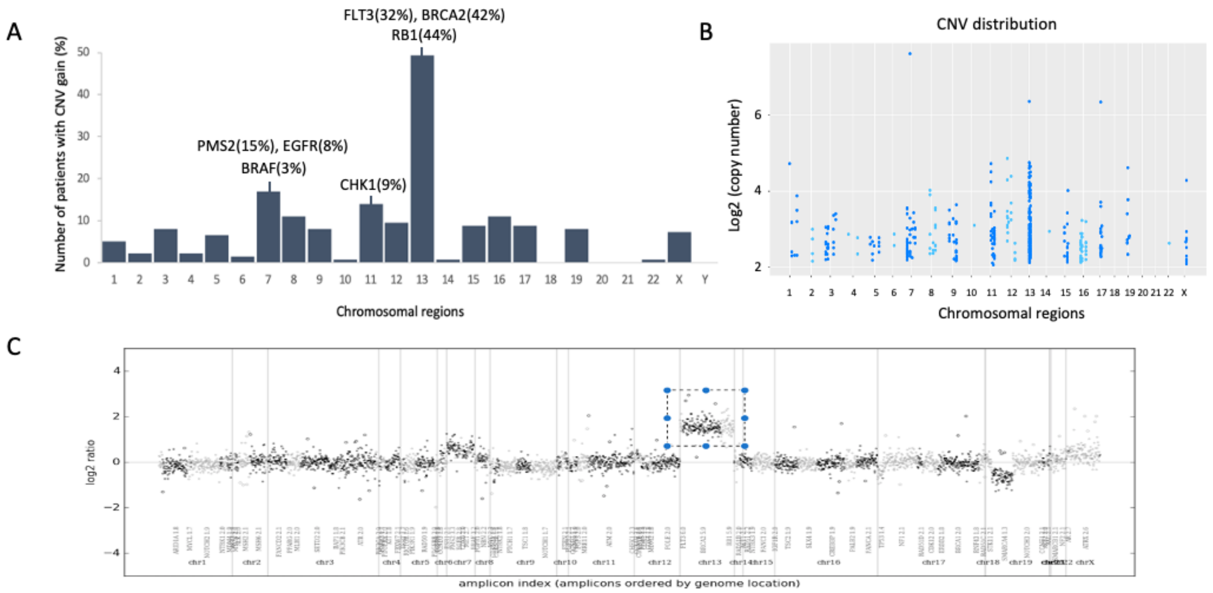

Figure S3: *Copy number variation in PM-CRC. (A) Percentage of patients with copy number gain in specific chromosomal regions. (B) Distribution of all copy number gains in chromosomal regions across all samples, showing all copy numbers above 4 (above log-value 2). (C) Integrative genomic viewer profile of copy number gains in chromosome 13 from one PM-CRC case.*

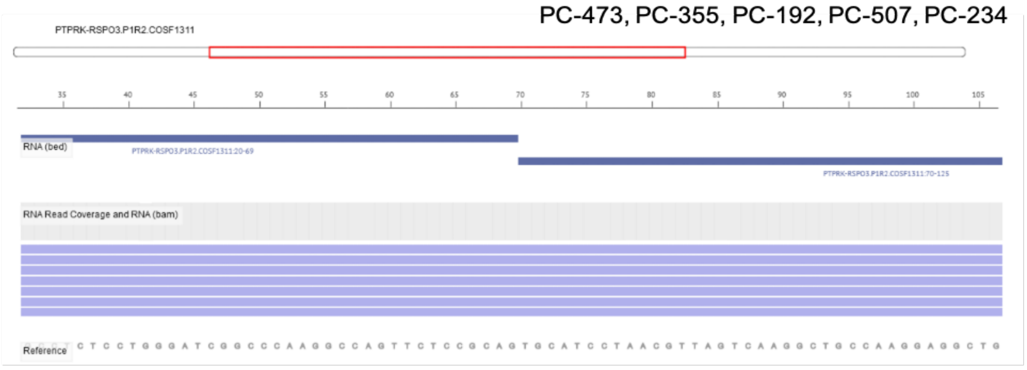
PC-473, PC-355, PC-192, PC-507, PC-234
PTPRK-RSPO3.P1R2.COSF1311
RNA (bed)
RNA Read Coverage and RNA (bam)
Reference

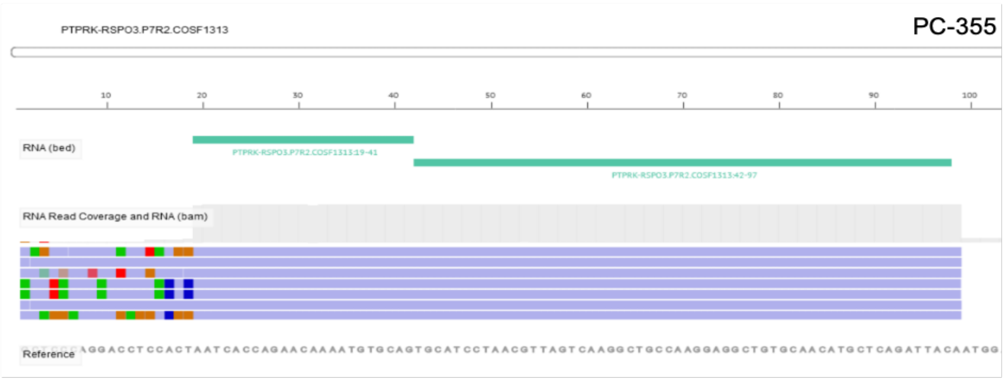
PC-355
PTPRK-RSPO3.P7R2.COSF1313
RNA (bed)
RNA Read Coverage and RNA (bam)
Reference

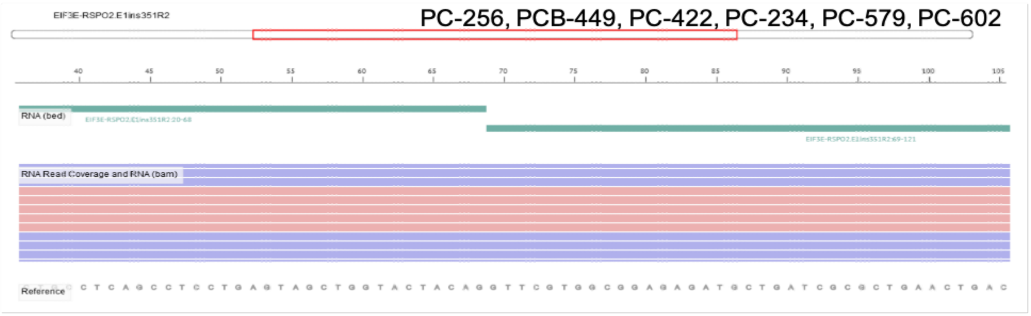
PC-256, PCB-449, PC-422, PC-234, PC-579, PC-602
EIF3E-RSPO2.E1ins351R2
RNA (bed)
RNA Read Coverage and RNA (bam)
Reference

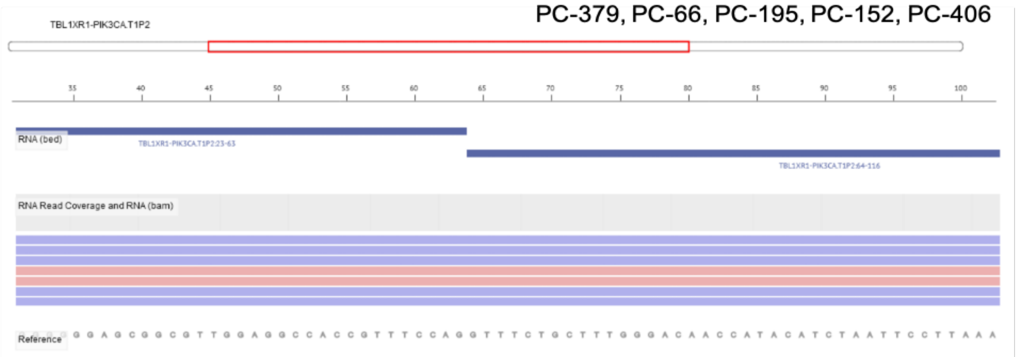
PC-379, PC-66, PC-195, PC-152, PC-406
TBL1XR1-PIK3CA.T1P2
RNA (bed)
RNA Read Coverage and RNA (bam)
Reference

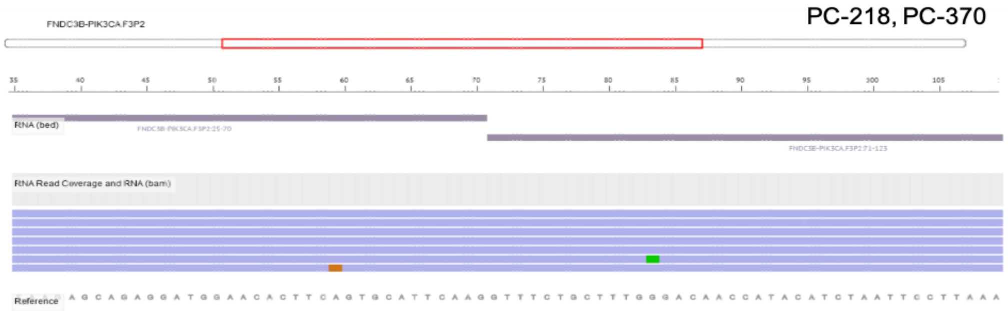
PC-218, PC-370
FNDC3B-PIK3CA.F3P2
RNA (bed)
RNA Read Coverage and RNA (bam)
Reference

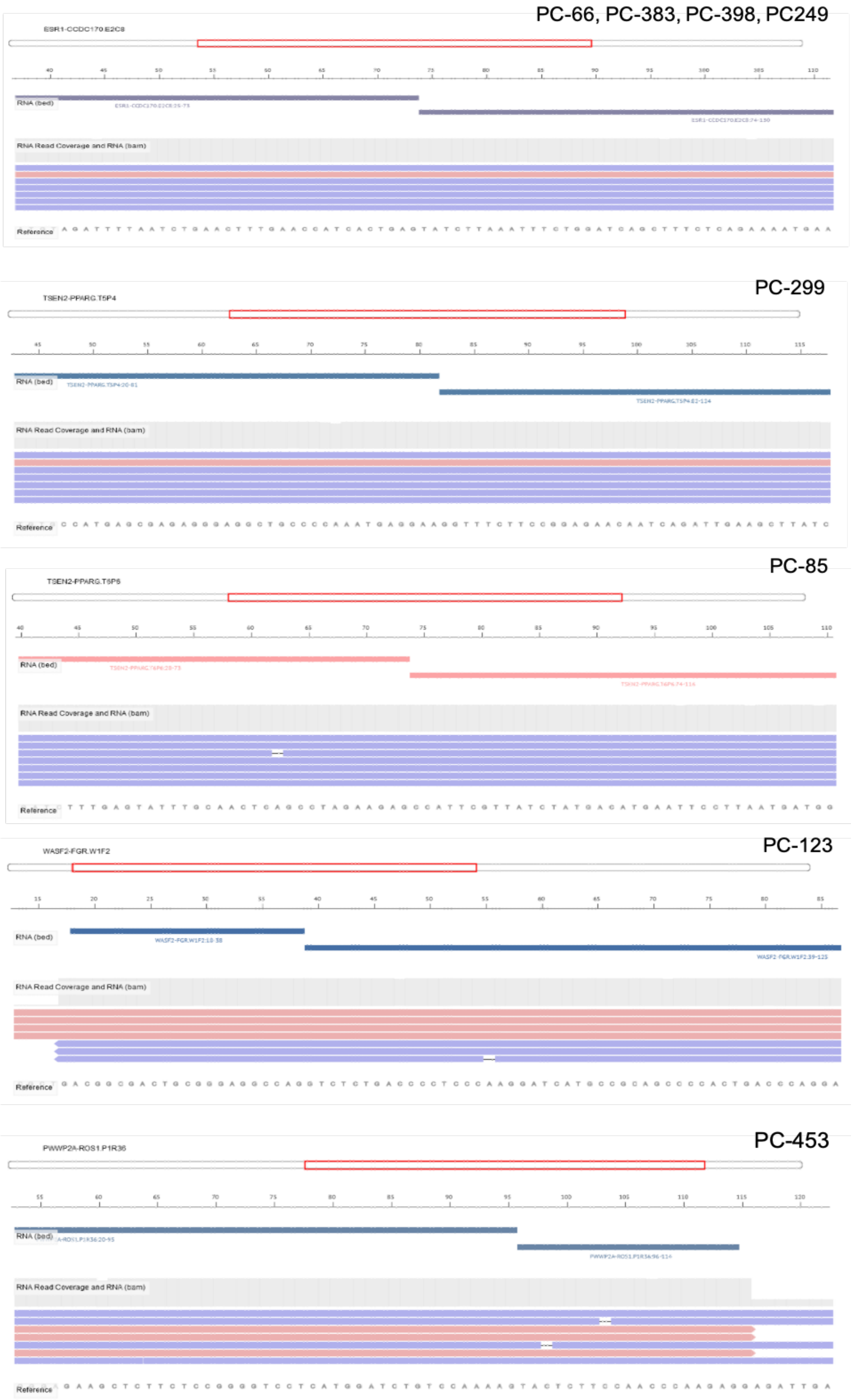

Figure S4: *Sequencing reads crossing the fusion breakpoint visualized through Integrative genomics viewer (IGV).*

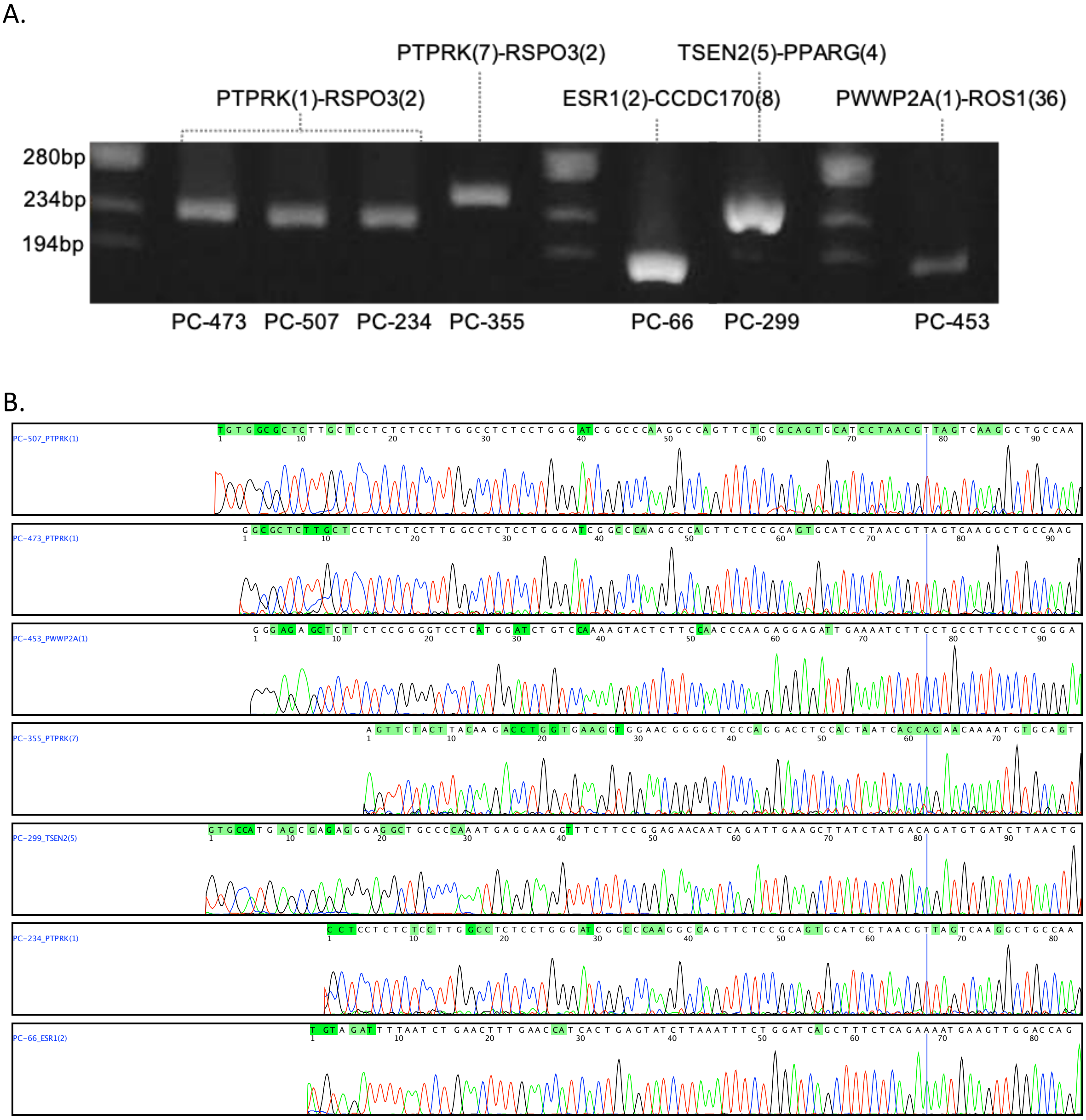

Figure S5: *Fusion gene validation. (A) Gel Doc image of fusion gene qPCR product and (B) Sanger sequencing of fusion genes across the fusion breakpoint*

Table S3: Univariable analysis (Cox regression) of overall survival in the total cohort (n=230)

		Overall survival, n=230		
Variabel		HR	95% CI	P-verdi
Age	Increasing	1,00	0,99-1,02	0,41
Gender	Female	Ref		
	Male	1,35	0,97-1,86	0,73
ECOG	0	Ref		
	1	1,01	0,59-1,72	0,98
Sidedness	App	Ref		
	Rt colon	1,34	0,77-2,33	0,30
	Left colon	0,99	0,52-1,66	0,83
	Rectum	1,03	0,48-2,20	0,94
T-status	Increasing	1,30	0,97-1,76	0,095
**N-status**	N0	Ref		
	N1	1,32	0,88-1,97	0,81
	**N2**	1,93	1,27-2,93	**0,002**
Histological	Low	Ref		
differentiation	Moderate	1,05	0,75-1,46	0,78
	Well	1,61	0,80-3,26	0,18
**PCI**	Increasing	1,09	1,07-1,11	**<0.01**
**CC-Score**	CC-0	Ref		
	CC-1,2,3	3,26	2,37-4,51	**<0,001**
BRAF	Mt	1,92	1,38-2,68	**<0,001**
NOTCH1	Mt	1,33	0.70-2,53	0,38
MSI	Mt	1,07	0,58-1,96	0,84
SMARCA4	Mt	0,517	0,19-1,41	0,20
TP53	Mt	1,20	0,88-1,64	0,24
PIK3CA	Mt	0,93	0,57-1,51	0,76
RNF43	Mt	1,57	0,90-2,75	0,12
ARID1A	Mt	1,60	0,70-3,69	0,27
KRAS	Mt	0,873	0,64-1,20	0,40
SLX4	Mt	0,75	0,30-1,84	0,52
NF1	Mt	1,51	0,78-2,92	0,22
APC	Mt	0,70	0,42-1,15	0,16
RB1	Mt	0,26	0,04-1,83	0,17
MSH6	Mt	3,32	1,44-7,66	**0,005**
BRACA1	Mt	0,81	0,30-2,21	0,68
CREBBP	Mt	0,66	0,24-1,79	0,41
ATRX	Mt	1,41	0,44-4,46	0,57
GNAS	Mt	1,22	0,65-2,32	0,54
BRCA2	Mt	1,44	0,74-2,78	0,28
NRAS	Mt	1,08	0,57-2,05	0,81
POLE	Mt	1,24	0,60-2,56	0,57
SETD2	Mt	0,79	0,36-1,71	0,55
FBXW7	Mt	0,87	0,46-1,65	0,67
ATR	Mt	0,79	0,29-2,14	0,64
PTCH1	Mt	0,76	0,31-1,88	0,55
MSH2	Mt	0,41	0,10-1,67	0,21
NOTCH2	Mt	0,25	0,04-1,83	0,17
ATM	Mt	1,01	0,56-1,82	0,97
RAD50	Mt	0,68	0,27-1,68	0,40
NOTCH3	Mt	0,95	0,41-2,19	0,90

SMAD4	Mt	1,86	1,23-2,79	**0,003**
FANCA	Mt	1,36	0,49-3,72	0,56
RSPO fusion	Mt	1,31	0,66-2,61	0,45
PIK3CA fusion	Mt	1,94	0,89-4,23	0,09
FLT3	Mt	2,03	0,50-8,25	0,32

Table S4: Univariable analysis (Cox regression) of overall survival and progression free survival in the CC-0 group (n=165)

		Overall survival, n=165			Progression free survival, n=164		
Variabel		HR	95% CI	P-verdi	HR	95% CI	P-verdi
**Age**	Increasing	1,01	0,99-1,02	0,41	1,02	1,01-1,04	**0,007**
Gender	Female	Ref			Ref		
	Male	1,26	0,82-1,89	0,30	1,21	0,83-1,75	0,324
ECOG	0	Ref			Ref		
	1	1,11	0,60-2,09	0,73	1,46	0,85-2,52	0,172
Sidedness	App	Ref			Ref		
	Rt colon	1,52	0,72-3,21	0,27	2,14	1,09-4,19	**0,027**
	Left colon	1,12	0,52-2,41	0,78	1,78	0,90-3,51	0,10
	Rectum	1,08	0,40-2,88	0,88	1,61	0,67-3,88	0,287
T-status	Increasing	1,33	0,92-1,93	0,13	1,05	0,75-1,49	0,761
**N-status**	N0	Ref			Ref		
	N1	1,35	0,84-2,19	0,22	1,32	0,86-2,04	0,21
	**N2**	1,97	1,20-3,23	**0,008**	1,52	0,97-2,39	0,07
Histological	Low	Ref			Ref		
differentiation	Moderate	1,12	0,73-1,70	0,61	1,13	0,77-1,66	0,532
	Well	1,49	0,58-3,80	0,41	1,38	0,54-3,49	0,50
**PCI**	Increasing	1,08	1,05-1,11	**<0.001**	1,05	1,02-1,08	**<0,001**
**BRAF**	Wt	Ref					
	Mt	1,92	1,26-2,93	**0,003**	1,77	1,19-2,63	**0,005**
**MSH6**	Wt	Ref					
	Mt	3,01	1,08-8,38	**0,035**	0,93	0,29-2,95	0,90
SMAD4	Wt	Ref					
	Mt	1,27	0,71-2,28	0,42	1,02	0,60-1,74	0,93
MSI	MSS	Ref					
	MSI	1,20	0,61-2,38	0,60	1,20	0,65-2,22	0,57
NOTCH1	Wt	Ref					
	Mt	1,09	0.44-2,68	0,85	1,60	0,81-3,17	0,176
SMARCA4	Wt	Ref					
	Mt	0,68	0,25-1,89	0,46	1,20	0,55-2,62	0,642
TP53	Wt	Ref					
	Mt	1,19	0,81-1,76	0,38	1,08	0,76-1,52	0,681
PIK3CA	Wt	Ref					
	Mt	0,97	0,52-1,82	0,93	1,20	0,68-2,13	0,529
**RNF43**	Wt	Ref					
	Mt	1,69	0,85-3,58	0,13	1,81	1,00-3,30	**0,051**
ARID1A	Wt	Ref					
	Mt	1,02	0,25-4,20	0,98	1,75	0,54-5,63	0,348
KRAS	Wt						

	Mt	0,94	0,63-1,39	0,75	0,92	0,65-1,31	0,65
SLX4	Wt						
	Mt	0,77	0,28-2,34	0,62	0,84	0,37-1,94	0,686
NF1	Wt						
	Mt	1,40	0,64-3,09	0,40	1,20	0,55-2,62	0,656
APC	Wt						
	Mt	0,72	0,38-1,37	0,32	0,74	0,39-1,41	0,361
RB1	Wt						
	Mt	0,67	0,09-4,84	0,70	1,03	0,25-4,17	0,97
BRACA1	Wt						
	Mt	0,76	0,24-2,45	0,65	0,83	0,34-2,06	0,689
CREBBP	Wt						
	Mt	0,62	0,19-1,99	0,42	1,00	0,43-2,30	0,99
ATRX	Wt						
	Mt	2,03	0,63-6,58	0,23	2,46	0,59-10,27	0,219
GNAS	Wt						
	Mt	1,16	0,43-3,14	0,78	1,71	0,63-4,67	0,296
BRCA2	Wt						
	Mt	1,38	0,63-3,04	0,43	1,33	0,66-2,68	0,426
NRAS	Wt						
	Mt	0,51	0,16-1,60	0,25	0,72	0,27-1,95	0,515
POLE	Wt						
	Mt	1,05	0,42-2,62	0,92	1,19	0,55-2,59	0,664
SETD2	Wt						
	Mt	0,64	0,23-1,77	0,39	0,94	0,45-1,95	0,858
FBXW7	Wt						
	Mt	0,89	0,41-1,91	0,76	0,69	0,34-1,41	0,31
ATR	Wt						
	Mt	0,86	0,27-2,74	0,79	0,62	0,27-1,43	0,258
PTCH1	Wt						
	Mt	0,58	0,18-1,89	0,58	0,81	0,33-2,00	0,645
MSH2	Wt						
	Mt	0,78	0,19-3,20	0,73	1,26	0,40-4,02	0,695
NOTCH2	Wt						
	Mt	0,32	0,05-2,34	0,26	2,06	0,64-6,63	0,225
ATM	Wt						
	Mt	1,29	0,63-2,66	0,49	1,33	0,70-2,54	0,39
RAD50	Wt						
	Mt	0,57	0,18-1,83	0,34	1,23	0,57-2,68	0,601
NOTCH3	Wt						
	Mt	1,00	0,39-2,53	0,99	0,82	0,38-1,80	0,627
FANCA	Wt						
	Mt	1,32	0,41-4,25	0,65	1,04	0,38-2,86	0,938
RSPO fusion	Wt						
	Mt	1,36	0,58-3,16	0,48	0,97	0,45-2,12	0,947
PIK3CA fusion	Wt						
	Mt	2,42	0,96-6,11	0,06	1,56	0,63-3,89	0,339
FLT3	Wt						
	Mt	2,69	0,37-19,64	0,33	1,27	0,18-9,14	0,812

Table S5: Top 10 up/down-regulated genes in *BRAF* mutated vs WT PM-CRC

Gene	Median log2FC	p_{adj}	Name
FEZF1	14.61	4.32E-10	FEZ family zinc finger 1
ANXA10	13.5	1.19E-07	Annexin 10
REG4	9.4	0.00029	Regenerating islet-derived type 4
ARX	6.48	0.001112	Aristaless Related Homeobox
SEMG1	6.48	0.04662	Semenogelin 1
TM4SF4	5.83	0.001536	Transmembrane 4 L Six Family Member 4
TFF1	5.49	0.00504	Trefoil factor 1
CTSE	5.38	9.13E-05	cathepsin E
TFF2	5.38	0.000129	Trefoil factor 2
SPINK4	4.73	4.32E-10	Serine Peptidase Inhibitor Kazal Type 4
PCDH20	-14.78	0.040195	Protocadherin 20
LY6G6D	-8.6	0.039415	lymphocyte antigen 6 family member G6F
SLC30A2	-6.87	0.016616	Solute Carrier Family 30 Member 2
FAM155B	-5.64	0.009381	NALCN Channel Auxiliary Factor 2
CPLX2	-5.23	1.05E-05	Complexin 2
VAV3	-5.21	0.013582	Vav guanine nucleotide exchange factor 3
CEL	-5.02	0.090428	Carboxyl Ester Lipase
NXPE4	-4.99	0.0255	Neurexophilin And PC-Esterase Domain Family Member 4
PTPRO	-4.78	0.000963	Protein Tyrosine Phosphatase Receptor Type O
MAP7D2	-4.63	4.72E-06	MAP7 Domain Containing 2

Table S6: Top 10 up/down-regulated genes in *BRAF* mutated vs KRAS PM-CRC

Gene	Median log2FC	p_{adj}	Name
AQP5	5.66	0.004505	Aquaporin 5
FAM72B	3.77	0.047581	Family With Sequence Similarity 72 Member B
FER1L6	3.74	0.000654	Fer-1 Like Family Member 6
DACT2	3.31	0.021198	Disheveled Binding Antagonist Of Beta Catenin 2
SUSD4	2.98	0.086143	Sushi Domain Containing 4
SCIN	2.88	0.01721	Scinderin
SLC9A7	2.61	0.02492	Solute carrier family 9 member A7
BTN1A1	2.53	0.068651	Butyrophilin Subfamily 1 Member A1
CPS1	2.49	0.000289	Carbamoyl-phosphate synthase 1
ABCA3	2.38	0.006632	ATP Binding Cassette Subfamily A Member 3
PPP2R2C	-5.86	0.021844	Protein Phosphatase 2 Regulatory Subunit B gamma
CCL24	-5.74	0.090642	C-C Motif Chemokine Ligand 24
KRT6A	-5.34	0.02851	Keratin 6A
PTPRO	-4.69	0.009659	Protein Tyrosine Phosphatase Receptor Type O

GRM8	-4.0	4.7E-06	Glutamate Metabotropic Receptor 8
TFCP2L1	-4.46	0.041803	Transcription Factor CP2 Like 1
RUBCNL	-4.2	0.038309	Rubicon Like Autophagy Enhancer
NOTUM	-3.6	0.000654	Notum, Palmitoleoyl-Protein Carboxylesterase
CAPN12	-4.08	0.079344	Calpain 12
IGFL4	-3.98	0.005094	IGF Like Family Member 4

Table S7: *Gene set enrichment analysis of BRAF mutated PM-CRC vs KRAS mutated and WT (grey, downregulated; green, upregulated)*

GO.ID	Description	p.Val	FDR	-logFDR	Comparison	Genes
KEGG:00190	Oxidative phosphorylation	0.0021	0.0021	2.68	KRAS	MT-CYB,MT-ND1,MT-ND4L,MT-ND2,MT-ND6,MT-ND5,ATP6V0A1
KEGG:04310	Wnt signaling pathway	0.0024	0.0024	2.61	KRAS, WT	AXIN2,NKD1,ZNRF3,FZD7,PLCB4,RNF43,TCF7,VANGL2,FZD6
KEGG:04723	Retrograde endocannabinoid signaling	0.0130	0.0130	1.89	KRAS	MT-ND1,MT-ND4L,MT-ND2,MT-ND6,MT-ND5,PLCB4
REAC:R-HSA-425407	SLC-mediated transmembrane transport	0.0370	0.0370	1.43	WT	SLC26A2,SLC4A8,SLC9A3,SLC6A6,SLC26A3,SLC30A2,SLC5A6,SLC5A1,SLC7A6
REAC:R-HSA-425393	Transport of inorganic cations/anions and amino acids/oligopeptides	0.0414	0.0414	1.38	WT	SLC26A2,SLC4A8,SLC9A3,SLC6A6,SLC26A3,SLC7A6
KEGG:04390	Hippo signaling pathway	0.0463	0.0463	1.33	KRAS	BMP4,TCF7,PPP2R2C,AXIN2,ID1,NKD1
KEGG:05415	Diabetic cardiomyopathy	0.0475	0.0475	1.32	KRAS	MT-CYB,MT-ND1,MT-ND4L,MT-ND2,MT-ND6,MT-ND5,PLCB4
KEGG:05016	Huntington disease	0.0608	0.0608	1.22	KRAS	MT-CYB,MT-ND1,MT-ND4L,MT-ND2,MT-ND6,MT-ND5,PLCB4,GPX2
KEGG:04714	Thermogenesis	0.0706	0.0706	1.15	KRAS	ACSL5,MT-CYB,MT-ND1,MT-ND4L,MT-ND2,MT-ND6,MT-ND5
REAC:R-HSA-168256	Immune System	0.0575	0.0575	1.24	KRAS	RSAD2,PRSS2,GBP4,CXCL10,BST2,IFIT1,TUBAL3,IFIT3,PAG1,IFI16,IRS1,BTN1A1,GBP1,IFIT2,HLA-DPA1,PELI2,MUC5AC,BTNL9
KEGG:00982	Drug metabolism - cytochrome P450	0.0462	0.0462	1.33	WT	ALDH3B1,MAOB,UGT2B7,CYP3A4
KEGG:05164	Influenza A	0.0269	0.0269	1.57	KRAS	RSAD2,PRSS2,CXCL10,HLA-DQB1,HLA-DPA1
KEGG:00140	Steroid hormone biosynthesis	0.0183	0.0183	1.74	WT	SRD5A3,UGT2B7,HSD17B2,CYP3A4
REAC:R-HSA-1280215	Cytokine Signaling in Immune system	0.0175	0.0175	1.76	KRAS	RSAD2,GBP4,CXCL10,BST2,IFIT1,IFIT3,IRS1,GBP1,IFIT2,HLA-DPA1,PELI2
REAC:R-HSA-909733	Interferon alpha/beta signaling	0.0015	0.0015	2.82	KRAS	RSAD2,BST2,IFIT1,IFIT3,IFIT2
REAC:R-HSA-913531	Interferon Signaling	0.0003	0.0003	3.49	KRAS	RSAD2,GBP4,BST2,IFIT1,IFIT3,GBP1,IFIT2,HLA-DPA1

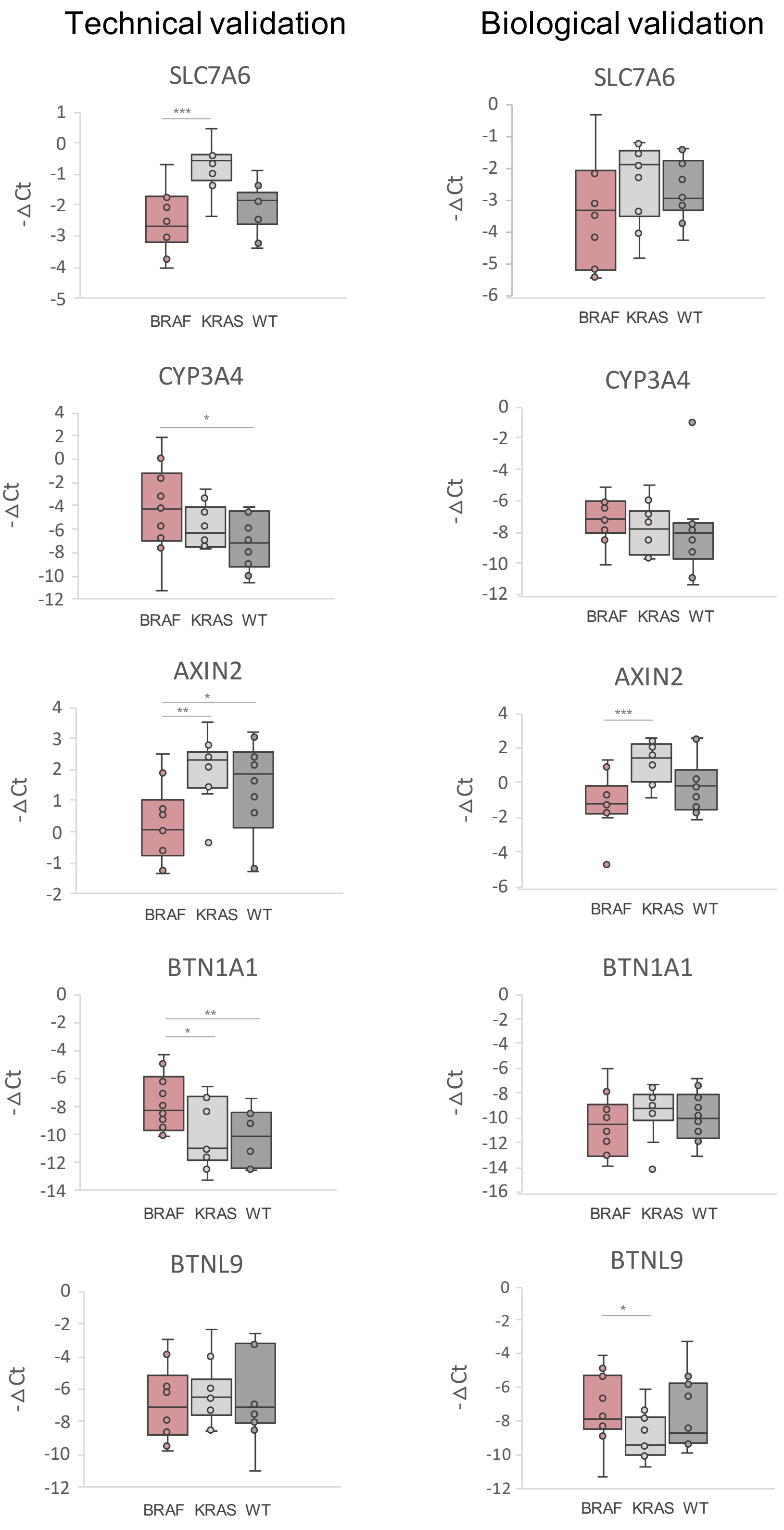

Figure S6: *Validation of gene expression results from RNA sequencing results with qPCR and comparative quantification. Technical validation of selected genes in the 30 samples that were subjected to mRNA sequencing (left panel). Biological validation of the same genes in supplementary samples with similar mutational profiles (n=30). ΔCt is the difference in gene expression level between the target gene and reference gene (YARS). Data behind the figure are provided in supplementary file 9*
